# Care beyond the hospital ward: understanding the socio-medical trajectory of herpes simplex virus encephalitis

**DOI:** 10.1186/s12913-017-2608-2

**Published:** 2017-09-12

**Authors:** Jessie Cooper, Ciara Kierans, Sylviane Defres, Ava Easton, Rachel Kneen, Tom Solomon, on behalf of ENCEPH-UK study group, Ruth Backman, Gus Baker, Nicholas Beeching, Rachel Breen, David Brown, Chris Cheyne, Enitan Carrol, Nick Davies, Sylviane Defres, Ava Easton, Martin Eccles, Robbie Foy, Marta Garcia-Finana, Julia Granerod, Julia Griem, Michael Griffiths, Alison Gummery, Lara Harris, Helen Hickey, Helen Hill, Ann Jacoby, Hayley Hardwick, Ciara Kierans, Michael Kopelman, Rachel Kneen, Gill Lancaster, Michael Levin, Rebecca McDonald, Antonieta Medina-Lara, Esse Menson, Benedict Michael, Natalie Martin, Manish Sadarangani, Andrew Pennington, Andrew Pollard, Julie Riley, Anne Christine Salter, Maria Thornton, Angela Vincent, Charles Warlow

**Affiliations:** 1 Division of Health Services Research & Management, City, University of London, London, UK; 2 Department of Public Health and Policy, University of Liverpool, Liverpool, UK; 3 Clinical Infection, Microbiology and Immunology, Institute of Infection and Global Health, University of Liverpool, Liverpool, UK; 4 Tropical and infectious diseases Unit, The Royal Liverpool and Broadgreen University Hospitals Trust, Liverpool, UK; 5 NIHR HPRU in Emerging and Zoonotic Infections, Institute of Infection and Global Health, University of Liverpool, Liverpool, UK; 6 The Encephalitis Society, Malton, North Yorkshire, UK; 7 Department of Neurology, Alder Hey Children’s NHS Foundation Trust, Liverpool, UK; 8 Department of Neurology, The Walton Centre NHS Foundation Trust, Liverpool, UK

**Keywords:** Encephalitis, Herpes simplex virus, Care inequalities, Narrative research, Qualitative, Health-work, Patient trajectories

## Abstract

**Background:**

Herpes simplex virus (HSV) encephalitis is a life-threatening infection of the brain, which has significant physical, cognitive and social consequences for survivors. Despite increasing recognition of the long-term effects of encephalitis, research and policy remains largely focused on its acute management, meaning there is little understanding of the difficulties people face after discharge from acute care. This paper aims to chart the problems and challenges which people encounter when they return home after treatment for HSV encephalitis.

**Methods:**

The paper reports on data from 30 narrative interviews with 45 people affected by HSV encephalitis and their significant others. The study was conducted as part of the ENCEPH-UK programme grant on *Understanding and Improving the Outcome of Encephalitis.*

**Results:**

The findings show the diverse challenges which are experienced by people after treatment for HSV encephalitis. We first chart how peoples’ everyday lives are fragmented following their discharge from hospital. Second, we document the social consequences which result from the longer-term effects of encephalitis. Finally, we show how the above struggles are exacerbated by the lack of support systems for the post-acute effects of encephalitis, and describe how people are consequently forced to devise their own care routines and strategies for managing their problems.

**Conclusion:**

The paper argues that in order to improve long-term outcomes in encephalitis, it is vital that we develop pathways of support for the condition beyond the acute hospital setting. We conclude by making recommendations to enhance communication and care for the post-acute consequences of encephalitis, to ensure those affected are fully supported through the chronic effects of this devastating disease.

## Background

Encephalitis is inflammation of the brain tissue caused by infection or autoimmune disease. Recent research has indicated that encephalitis is more prevalent than previously considered, with an annual incidence of between 5 and 8 in 100,000 [1]. In the UK, the most commonly identified infectious cause of encephalitis is the herpes simplex virus (HSV), which affects around 1 in 250,000–500,000 per year [2, 3].

In the past three decades, there have been marked improvements in mortality rates from HSV encephalitis, due to early treatment with the antiviral drug, acyclovir [4, 5]. While the acute onset of the condition is now highly treatable, survivors are usually left with neuropsychiatric sequelae. This acquired brain injury can have myriad physical, cognitive and social consequences for those affected and their significant others [4, 6, 7]. For example: adults and children affected by HSV encephalitis have increased risk of developing epilepsy and cognitive problems, including short-term memory loss and difficulties with language and communication, known as aphasia. In children, encephalitis is linked to developmental delays; and poor mental health, such as depression and anxiety, is commonly reported by those affected by all types of encephalitis [8–11]. A small body of qualitative research has also highlighted the profound impact on the identity of those affected, such as the ‘loss of self’ experienced as a result of memory problems [6, 12]. Unsurprisingly, therefore, encephalitis has a long-term, adverse effect on quality of life for the majority of people who survive the illness [13].

However, despite recognition of the chronic effects of HSV encephalitis, research and policy remains largely focused on acute management of the condition [6, 7]. For example, the recently published National Clinical Guidelines for the Management of viral encephalitis cover the initial management of patients with suspected encephalitis, and treatment following a diagnosis of viral encephalitis [3, 14]. Whilst these guidelines make recommendations about ensuring there are arrangements for out-patient follow-up and a plan for rehabilitation at discharge, there is little understanding of how this support works in practice, or the challenges which patients face once they return home. In order to fully understand and appropriately support the long-term outcomes of HSV encephalitis, it is crucial that we examine what happens when people leave hospital following treatment for the condition.

This paper charts the challenges and problems people encounter after acute HSV encephalitis. Drawing on narrative interview data, it shows how the problems experienced post-discharge are exacerbated in the absence of clear pathways of care for the chronic effects of HSV encephalitis. The paper then proposes a series of recommendations for improving the longer-term support for people affected by encephalitis.

## Methods

This paper uses narrative interview data from a project called the *End User Experience Study*, part of a National Institute for Health Research (NIHR) applied research programme grant on *Understanding and Improving the Outcome of Encephalitis* (ENCEPH-UK). The *End User* study aimed to develop a detailed understanding of HSV encephalitis and its management from the perspective of people affected by the condition, as well as their significant others. Full details of the programme can be accessed on the ENCEPH-UK website [15]. The programme was approved by the National Research Ethics Service (NRES), East Midlands Committee (11/EM/0442).

Narrative research is a form of qualitative inquiry which uses in-depth interviews to focus on the remembered account, in order to explore how people recall, account for, and make sense of past events and actions [16]. A narrative methodology enables insight into the experience of illness, as well as allowing for individual accounts to be understood with reference to the wider socio-cultural contexts which underpin and construct these experiences, in this case, systems of care in the UK [17–19]. Narrative inquiry has long been acknowledged as an important methodology for uncovering issues in health and healthcare, which cannot be accessed by more conventional means, such as quantitative surveys and clinical measurements [20, 21]. This approach is therefore well suited to providing access to experience, and its patterning, in the context of post-acute encephalitis.

Participants with HSV encephalitis were recruited to the *End User Experience study* from the main ENCEPH-UK cohort studies. These were made up of two groups of participants recruited from 60 hospitals across the UK: 1) a retrospective cohort who had encephalitis at any time between 2005 and 2012, and 2) a prospective cohort of patients, recruited at the point of having suspected encephalitis in the hospital sites and interviewed between 3 and 6 months after discharge from hospital. Additional retrospective participants were recruited through *The Encephalitis Society*, a partner on the ENCEPH-UK programme. Children in the ENCEPH-UK programme were recruited via the Childhood Meningitis and Encephalitis study (UK-ChiMES), a collaborative study with the University of Oxford [15].

Participants were first contacted by phone or email and were sent an information sheet about the study. In total, 30 narrative interviews were conducted with 45 participants in their homes (17 interviews with 26 participants from the retrospective cohort and 13 interviews with 19 participants from the prospective cohort). Since those affected by encephalitis often struggled to remember details of their acute illness, many chose to be interviewed with a relative or friend, meaning these accounts were co-produced by patients and their significant others. Ethical constraints meant that the parents of children under the age of 16 were interviewed, rather than the child (*n* = 5). Additionally, interviews were done solely with relatives for two adult cases: one in which the patient had died, and in the other, the patient had acquired severe neurocognitive problems, meaning she was unable to participate. One participant from the prospective cohort subsequently withdrew from the study, making 29 transcripts available for the final analysis. Table 1 describes the demographics of our participants, including the length of stay in hospital and their employment status after encephalitis.

**Table 1:** Participant characteristics and interview details of participants with HSV encephalitis

Person with HSV encephalitis	Age at interview	Gender M/F	Timing of interview post-discharge	Interview details	Duration of stay in hospital (days)	Destination on discharge	Employment status at time of interview
Retrospective Cohort							
1	45	M	6 years	Interviewed with partner	22	Home	No longer working: receiving Disability Living Allowance
2	47	F	7 Years	Interviewed with mother	211	Home	No longer working: receives income from previous employer’s insurance
3	43	M	6 years	Interviewed with partner	20	Home	No longer working: receiving Disability Living Allowance
4	58	M	5 years 10 months	Interviewed with wife	30	Home	Employed: took a role with fewer responsibilities after being made redundant.
5	15	M	1 year 11 months	Interview conducted with the parents	63	Home	N/A – in school
6	62	F	5 years 9 months	Interviewed alone	48	Home	No longer working: sold her business
7	68	F	7 years 1 month	Interviewed alone	16	Home	Took early retirement
8	55	F	5 years	Interviewed with friend	15	Home	Took early retirement
9	36	M	1 year	Interviewed with wife	10 (continued acyclovir at home)	Home	Employed: took a role with fewer responsibilities post-encephalitis
10	5	M	5 years 7 months	Interview conducted with the child’s mother	37	Home	N/A – in school
11	56	F	3 years 6 months	Interview conducted with husband	126	Neuro-rehabilitation	No longer able to work
12	20	F	5 years 10 months	Interviewed alone	39	Home	Student (university)
13	34	F	4 years 4 months	Interviewed with partner	21	Home	No longer working: receiving Disability Living Allowance
14	55	F	7 years 7 months	Interviewed alone	12	Home	Took early retirement
15	6	M	3 years	Interview conducted with the child’s father	42	Home	N/A – in school
16	33	M	1 year 2 months	Interviewed with Mother	25	Home	Employed: returned to work
17	61	F	6 years 5 months	Interviewed alone	58	Home (offered rehabilitation but declined)	Took early retirement
Prospective Cohort							
1	69	M	5 months	Interviewed alone	45	Rehabilitation	Already retired
2	58	M	5 months	Interviewed with wife	24	Home	Self-employed: receiving Employment Support Allowance
3	27	M	3 months	Interviewed alone	52	Rehabilitation	Employed: returned to work
4	61	F	3 months	Interviewed with husband	117	Neuro-rehabilitation	Already retired
5	67	M	3 months	Interviewed with wife and daughter	40	Home	Already retired
6	77	F	5 months	Interview conducted with husband and son (patient died)	122	Nursing home	Patient died
7	35	M	4 months	Interviewed alone	18	Home	Employed: returned to work
8	58	F	6 months	Interviewed alone	60	Rehabilitation	Employed: returned to work
9	75	M	3 months	Interviewed with wife	52	Rehabilitation	Already retired
10	63	F	4 months	Interviewed with sister	29	Home	Never worked
11	6 months	F	3 months	Interview conducted with the child’s mother	3 (treatment continued with home care team)	Home	N/A
12	2	M	3 months	Interview conducted with the child’s mother	25	Home	N/A

Written consent was taken from participants before each interview. The interviews lasted between 20 min and three and a half hours and were digitally recorded and transcribed. All the accounts were anonymised for identifying features during transcription, including assigning participants with pseudonyms. The interviews covered topics about participants’ experiences of: diagnosis, care received in hospital, everyday life post-hospitalisation, and post-discharge care (see End User Study narrative interview guide, in supplementary material).

While the narratives demonstrated a diverse range of experiences, the analysis was particularly concerned with how people made sense of their experience, and the ‘structural commonalities’ across accounts, such as how peoples’ narratives emphasised, and were shaped by, the absence of systems of support after discharge from hospital [22, 23]. In order to examine, in-depth, the individual accounts and the commonalities between them, we incorporated a narrative analytic approach with a more general thematic analysis [16]. This dual process involved, first, conducting a structural narrative analysis on the data, which focused on characterising the organisation of the narratives, in relation to how events were described and interpreted within the accounts. The categories generated from the narrative analysis were then used as a framework for the thematic analysis. This was concerned with identifying and interpreting the patterns across accounts, in terms of the common challenges which people encountered after being discharged from hospital and how they responded to these; for example, the struggles people experienced with gaining access to specialist care, and the actions they took as a result. The thematic analysis was managed using the qualitative data management software, Nvivo 10. This dual analytic approach meant that we were able to characterise the conditions which shape the individual experiences across the narrative accounts as a whole [24].

To illustrate the findings we have presented the data in the form of in-depth cases, relating to three different participants. The use of cases is a commonplace way to present narrative data, and we have successfully employed this approach in a previous publication on this study [18, 24]. If we relied on presenting carved-up interview quotations, as is standard in other forms of qualitative research, this would not allow for insight into the common structuring of our participants’ accounts, for example, how peoples’ experiences of the chronic effects of encephalitis were underpinned by the absence of clear pathways of post-acute care [23]. It is important to emphasise, therefore, that the three cases that were selected, and which are presented below, were chosen for the way in which they depict the structure of peoples’ experiences, most typically, across the dataset as a whole.

## Results

Below, we present the cases of Simon, Joanna, and Joan. The cases reveal the problems which people commonly encounter after HSV encephalitis, whilst also showing how these challenges are underpinned by the lack of coherent provisions of care for the chronic effects of encephalitis.

### Simon

*Simon is a man in his 40s with HIV. He was diagnosed with HSV encephalitis 6 years prior to being interviewed. Before being affected by encephalitis, Simon worked as an illustrator. Simon was interviewed alongside his partner, Pete.*

Simon was diagnosed with HSV encephalitis after being successfully treated for lymphoma. While Simon and Pete felt that the care received in hospital was good, they described how problems started once Simon was discharged from hospital. Upon his return home, Simon recounted how he “didn’t understand what the hell was going on”: he was unable to drive and could not understand why he was so tired and forgetful. Pete was afraid to leave Simon’s side at this time, since Simon’s memory and concentration was so badly affected that he couldn’t remember how to get to familiar places, even crossing a road proved difficult. Both men struggled to understand these difficulties, and Pete felt let down by the absence of information provided by the hospital about what to expect after encephalitis. Pete had been so relieved that Simon had survived encephalitis that it took some time to realise that things were not returning to “normal”.

Simon’s problems with memory and concentration had serious consequences for his everyday life. He was unable to continue working as a freelance illustrator because he struggled to get to grips with the organisational side of the business, in particular with meeting deadlines and communicating with clients. Eventually, he applied for Disability Living Allowance, something he recalled as a “depressing” process, since he had to document all the problems that his memory issues caused, such as forgetting what he has done the day before; forgetting where he left things; finding conversations difficult to follow; and experiencing fatigue. Simon described his memory as fragmented, like “looking at the world through a broken window”. He explained, “You can't actually make it [world] out because it's like someone has smashed the glass”. Over the 6 years since his diagnosis, Simon has developed elaborate strategies to cope with the practical effects of memory loss. To do this, he takes lots of photographs so he can remember each day; he avoids visiting large supermarkets, preferring to shop at smaller stores; he makes lists, and fastidiously plans out routes on google maps in order to “get rid of as many variables” as possible which may cause him difficulties.

Simon’s problems were further intensified by his long struggle to get support for his memory problems. Upon his initial discharge from hospital, he was told that he would receive an appointment with a consultant and that he should visit his GP if there were any immediate problems. Simon initially received some general counselling through the hospital; however, he found this unhelpful because it wasn’t specialised enough to deal with his memory problems. He then turned to his HIV doctor for advice, who referred him for neuropsychology testing. Simon had hope that this would lead to some solutions, however, the service was not what he expected and he was disappointed when the neuropsychologist told him that his memory would not improve. After seeking advice from *The Encephalitis Society*, Simon and Pete tried to get a referral to the brain injury charity *Headway* for specialist counselling, but were told that treatment was contingent on funding from their Local Authority. After a year of waiting, Simon wrote to his local MP to ask for help securing the funding. During this time he also received the offer of cognitive behavioural therapy (CBT) through the hospital neurology department. After 6 years of feeling like he had been left “festering” following encephalitis, Simon started a course of CBT with a therapist. He explained that he felt like he was finally “teamed up with the right person”, who tailored the programme to meet his needs. This support has helped him improve his memory and concentration, enabling him to go Christmas shopping alone, and become more independent in his daily life.

### Joanna

*Joanna is a woman in her 50s who was treated for HSV encephalitis 5 years before being interviewed. Joanna had been employed in education before her diagnosis and was interviewed alongside her friend, Ruth.*

Joanna was discharged home after spending two weeks in intensive care following a diagnosis of HSV encephalitis. Joanna spent much of the first few months after hospital in bed and described how she experienced “horrendous head pains” during this time. She was unable to turn her head without feeling dizzy and could not understand what was wrong with her. Ruth took on responsibility for caring for Joanna during this time, and recalled her exhaustion from worrying about Joanna, who she was unable to leave alone for even an hour. As Joanna started to physically recover, she became aware that things were “not working so well”. Her short-term memory was “shot to pieces”, and she had difficulties with her speech, meaning she could not find the right words for things. The effects of her memory loss also meant Joanna had difficulties carrying out everyday tasks, such as writing and making a cup of tea. As a consequence, she found herself disconnected from her old life as a successful professional. What had once been familiar activities, like writing, were now experienced as strange and disorientating. After 9 months recovering at home, Joanna realised she would not be returning to work. On the advice of an occupational health doctor, and after months of “absolute hell” filling out disability benefits forms, Joanna received early retirement, sold her flat and moved in with Ruth. This had practical and financial consequences for Ruth, who gave up her job as a professional carer to look after Joanna. To do this, she attempted to receive Carers Allowance as Joanna’s full-time carer. However, the process of applying for state support was both frustrating and, ultimately, fruitless, since she did not meet the required criteria.

After her discharge from hospital Joanna was given appointments with a neurologist and neuro-psychologist, who told her the encephalitis had caused permanent brain damage. Joanna found this important to hear, since it meant she wasn’t falsely expecting to make a full recovery. Despite this diagnosis, Joanna attempted to find help for her speech and memory problems. After seeking information from *The Encephalitis Society*, she requested to see a speech therapist for her difficulties with word-finding. However, her first appointment left her in tears on being told there was little wrong with her. Needing the extent and seriousness of her problems to be fully recognised, Joanna then sought help from her GP, who, in Ruth’s words, “took things seriously” and referred her to a neurological rehabilitation centre. Here Joanna was given a full description of what had occurred to her and strategies she could put in place to support her everyday tasks, such as cooking and getting the bus to visit her grandchildren. Joanna also made contact with the charity, *Headway*, where Ruth received respite services and Joanna saw a speech therapist. As a result, Joanna and Ruth have been able to develop tactics for dealing with Joanna’s difficulties. These include: closing her eyes to help her process sounds; meticulously planning out routes on public transport; using rolodex cards to work through the stages of a recipe; and keeping people in her line of vision during conversation to avoid becoming disoriented. Joanna sees these strategies as huge achievements, especially in light of her initial struggles to gain professional recognition and support for her problems.

### Joan

*Joan is a woman in her late 50s who was discharged from hospital 6 months prior to the interview. She is a practice nurse, who, at the time of interview was undergoing a phased return to work. Joan is married and was interviewed alone.*

Joan was treated for HSV encephalitis in hospital for 2 weeks before she was transferred to an in-patient rehabilitation unit, where she stayed for two months. She explained her distress when she first tried to get dressed in the rehabilitation centre and realised that she was unable to put her clothes on or brush her hair: “I knew I was doing it wrong but I didn’t know how to put it right”. What had been previously simple tasks, done without thought, were now disrupted by an unruly body, unable to do things in the right way. At the rehabilitation centre, her occupational therapist reassured her about the difficulties she was experiencing: “she said ‘don’t get upset about it, it’s just the processing of your brain and it will come back, it just takes time’ […] because I had got myself into such a state, and you are thinking one minute you are fine and the next you are 58 years old and can’t put your trousers on”. The therapist’s explanation enabled Joan to better understand her situation and that her inability to dress herself was not just because she was “being silly”. Joan described the care she received in the unit as “wonderful”: here she was given support to re-learn tasks such as cooking, and was given help with improving her memory.

Returning home from rehabilitation was, at first, very scary for Joan. Activities like going up and down the stairs were daunting, and Joan’s husband would stand behind her whilst she made her way on the stairs, to ensure she was safe. Joan set herself the initial challenge of walking for 10 min each day to reach her local supermarket. Over time she measured her improvement by the fact that she was no longer exhausted after this activity. Although she was still waiting for an appointment to see a neurologist at the time of her interview and gets very tired, she was “happy” with her situation and has a supportive GP who she is able to turn to for advice. Joan was off work for 3 months after her return home. In the weeks preceding the interview Joan had a phased return to her job at a GP surgery, going in for 3 half days a week (she had previously worked 4 full days). She expressed relief, feeling that her life was returning to how it was before encephalitis: “when I first went back I wasn’t given clinics of my own […] I was working with the other nurses because they have got to make sure the patients are safe with me and I am safe with the patients […] but it was just so lovely to be back at work and it felt as if that was the final piece in the jigsaw you know, my life is now back to normal”.

## Discussion

The cases presented above show the typical challenges which people encounter after they are discharged from hospital following treatment for HSV encephalitis. The narratives of Simon and Joanna also reveal how such difficulties are made worse by the absence of systems of care for the chronic effects of encephalitis. In the sections which follow we characterise these challenges in three ways: firstly, we chart how people’s everyday lives are fragmented following their discharge from hospital; secondly, we document the social consequences which result from the longer-term effects of encephalitis. Finally, we show how the above struggles are exacerbated by the lack of support systems for the post-acute effects of encephalitis, describing how people are consequently forced to devise their own routines of care and strategies for managing their problems. These findings are then used to generate recommendations for improving the post-acute support for people affected by encephalitis.

### Fractured lives: Experience after discharge from hospital

In the first few weeks and months following discharge from acute care, most of our participants [27/29 (93%)] experience a spectrum of disorienting and painful symptoms, which include: extreme headaches and tiredness, meaning much of the first weeks and months, post-discharge, are spent in bed; problems with walking, balance, and coordination; language difficulties, including the incapacity to locate the correct words when speaking and writing; and memory loss, forgetting what has occurred on a daily or hour-to-hour basis. The co-ordinates of speech, memory, and movement, which are part of the taken-for-granted structuring of life, are therefore altered and the daily routines which they help to stabilise become fractured. For the majority of our participants [24/29 (83%)], this means that that re-entry into the basic routines of ‘normal life’ is experienced as strange and overwhelming. Taken-for-granted, embodied actions, such as washing, dressing, walking and talking are profoundly altered, meaning that other purposeful activities, like crossing the road, cooking, and driving become difficult and unfamiliar. These problems also mean that the daily lives of significant others are overtaken by the requirement to support these functions: to care for people who can no longer manage the activities of independent living. This is an exhausting process for those forced to care for their relatives around the clock, and who have to adjust their own working lives to facilitate a new role as carer.

### Fractured social worlds: Chronic effects of encephalitis

For all participants in the retrospective cases [17/17 (100%)], these difficulties are not short term: there is no ‘full’ recovery from the effects of encephalitis. Over time, the consequences of having encephalitis becomes pronounced, and incorporates, in addition to the problems outlined above: seizures, with some participants receiving a diagnosis of epilepsy; general fatigue and difficulties concentrating on tasks; mental health problems; and behavioural issues, such as aggression. Although these problems are disparate and experienced to varying degrees for each person, they have similar consequences across all the cases, in that they re-shape people’s everyday social worlds in two inter-related ways: 1) *change in material circumstance*, which impacts on employment, welfare entitlements and living circumstances. Most people [10/17 (59%)] are unable to return to work, and some people have to change jobs [3/17 (18%)]. For those who cannot return to work, they are forced to apply for welfare benefits or take early retirement (see Table 1). Changes in employment status also have serious financial implications, with individuals and families struggling to maintain former lifestyles. In some cases [4/17 (24%)], like Joanna’s, the combined changes in financial circumstances and difficulties with managing everyday routines mean that people are forced to alter their living arrangements, for example by downsizing, or moving in with family. *2) Adjusting inter-personal relationships*. Peoples’ ongoing difficulties lead to major adjustments in the role and nature of their relationships with their significant others. The day-to-day work involved in domestic routines, such as cooking, and taking children to school, becomes largely the responsibility of other family members. These shifts in responsibility mean that the nature of relationships between people affected by encephalitis and their significant others changes: spouses, older parents, friends, etc. become carers, with varying levels of responsibility. In some cases [5/17 (30%)], like Ruth, significant others also give up their own jobs to act as full-time carers for the person affected. In these cases, the financial blow is doubled, since the employment status for both the person affected and their significant other is altered by encephalitis.

The cases in our study therefore highlight the situated realities and serious shifts in peoples’ social worlds (financial, practical, relational) as a result of encephalitis. While the chronic effects of encephalitis and other acquired brain injuries have been well laid out in the research literature, these are usually documented in isolation of the wider social contexts within which peoples’ difficulties play out [6, 25–27]. As we show below, the consequences of encephalitis are exacerbated by the absence of a formal support system for the post-acute care of the condition.

### Searching for support: Work to manage the chronic effects of encephalitis

The cases of Simon and Joanna reveal the lack of pre-defined care pathways and services for dealing with the chronic effects of encephalitis, as described by our retrospective participants. Below, we map out the various struggles involved in obtaining information and formal care around encephalitis, and chart the work which people do to construct their own improvised systems of support for managing their difficulties.

#### The importance of information and support

Fundamental to the need for support after encephalitis is the provision of information about the condition and its consequences. Participants in the study expected to return to ‘normal’ after their treatment in hospital, meaning that patients and their significant others subsequently struggle to fathom why they, or their loved ones, are unable to reintegrate back into their daily lives. The inability to make sense of their situation is attributed by some [5/17 (30%)], like Simon, to being discharged from hospital without an adequate understanding of what has happened to them or their relative, and the problems they are likely to encounter once they return home. Moreover, on their discharge from hospital most people are told that they will receive an appointment with a consultant and to visit their GP with any problems. In other words, after leaving the intensive support of the hospital ward, people return home without a prefigured plan for support, or the care which is needed to help them deal with their newly fractured lives.

The confusion which people experience post-discharge is therefore underpinned by the reality of going home without an understanding of what has happened or, most importantly, what was to come. Without this knowledge, those affected and their significant others pursue their own understanding about encephalitis and its care. This includes researching encephalitis and available forms of support on the internet, and contacting charitable bodies, like *The Encephalitis Society*. However, the search for information is only the start of people’s journey to find support for their difficulties, as we describe below.

#### Journeys through care

Without any plan for support, many people in the retrospective study [11/17 (65%)] have no option but to seek out care services to help deal with their problems. Attempts to obtain care, however, involve numerous complications. These include: i) *having their needs taken seriously*. People have trouble with gaining recognition from health professionals that problems, such as speech difficulties, memory loss, and so on, warrant attention and require formal sources of support. While we have previously documented how patients failed to have their concerns taken seriously by health professionals during the acute onset of encephalitis, our findings here show this process continues into the chronic trajectory, indicating an overarching lack of recognition for this condition and its consequences, particularly among health professionals [24]. ii) *Improvising Care:* in their search to gain formal sources of support, people travel between, and are referred to myriad different services across the NHS, charitable bodies, and private organisations. The services travelled through include: neurology, psychiatry, psychology, speech therapy, memory clinics, brain injury charities, like *Headway*, private counsellors, and alternative therapists. In particular, journeys through care occur when people feel that existing services are unable to meet their needs, when professionals lacked understanding of the nature of their problems, as detailed above, and when professionals communicate they are not specialised enough to deal with the person’s problems. In turn, people are forced look for alternative options for care, often by revisiting their GP to ask for further referrals. When people are unable to find help within the NHS, some draw on other resources by pursuing privatised forms of care, such as counselling, which has obvious implications for the already compromised financial situation of people after encephalitis.

In travelling through and seeking out care services, people formulate their own improvised care pathways: work which is essential for creating a system of support which is not easily forthcoming [28]. That patients are forced to create their own care pathways ostensibly adds to the vulnerability of people experiencing life-altering problems, like memory loss. This raises questions about whether lack of access to appropriate care potentially contributes to differing outcomes in encephalitis. For the small number of people in the study [6/29 (21%)] who received structured rehabilitation, like Joan, their narratives often tell a different story of ‘recovery’ in two ways: i) rehabilitation is highlighted as important for aiding peoples’ ability to understand the problems they experience after leaving hospital. Being given explanations for why they struggle with routine tasks, like washing, means that people are better able to make sense of their situation. This contrasts with the experience of others, who return home unable to understand the nature of their problems; ii) The care which is received during rehabilitation, such as having help to re-learn activities like cooking, enables people to re-orient themselves to their everyday lives upon their return home. Focused rehabilitation therefore facilitates a more supported transition between the hospital and the home.

#### Improvised self-help strategies

In addition to sourcing appropriate medical care, participants also have to devise strategies to manage their difficulties in the context of their everyday routines [11/17 (65%)]. This work involves harnessing everyday materials and technologies, such as diaries and mobile phones, which act as aides for memories which no longer function in the way they used to. These strategies include: using diaries and taking photographs to remember participation in activities that people would otherwise forget; mobile phones are set with reminders for routine tasks; new routes are planned out with meticulous detail using tools such as google maps; post-it notes are stuck on household surfaces to jog people’s memory about the location of household objects or remind them to, for example, turn off the gas after cooking; strategies are devised for getting through tasks, such as cooking a recipe, as illustrated by Joanna’s use of rolodex cards; and crosswords and puzzles are practiced daily, in attempts to improve memory and re-establish lost language.

In lieu of adequate support systems for the chronic effects of encephalitis, those affected are thus forced to cobble together their own ad-hoc methods and improvised pathways in attempts to establish their own coherency of care [28, 29]. Research in the social sciences has long documented the ‘health-work’ and ‘labour’ which goes into managing chronic illnesses within the home [30–32]. However, these concepts usually refer to patient work which is organised by health professionals and directed around treatment regimes. In the context of HSV encephalitis in the UK, it is clear that patients are poorly provided with the knowledge or resources to inform their self-management. As a result, the management for the post-acute effects of encephalitis becomes largely, if not wholly, outsourced to being the responsibility of patients and their social networks.

### Recommendations from the findings

This study has shown that improving outcomes in encephalitis cannot be achieved by virtue of focusing on the acute stages of encephalitis alone. While the Introduction of National Clinical Guidelines have been vitally important in enhancing early diagnosis and treatment of viral encephalitis, more needs to be done to ensure that encephalitis care continues beyond the acute hospital setting, and there are equal opportunities for the receipt of this care.

The National Service Framework currently provides a template for the long-term care of people with neurological conditions, including the recommendation that everyone should have access to rehabilitation [33]. However, findings from this study show that, in practice, this is not the case for our participants, who experience a lack of communication about what to expect after encephalitis and fragmented provision of services. We therefore recommend that, first, all patients should be provided with, or signposted to, information about encephalitis and its effects, such as the resources provided on the website of *The Encephalitis Society* [34]. This would enable those affected and their significant others to gain an understanding of what has occurred and the forms of support which are available to them. Second, there is urgent need for the production of an integrated care pathway to cover the chronic consequences of encephalitis. This pathway should specify what aftercare can be provided to people post-encephalitis, and supply information and links to resources about its potential sequelae. This would ensure that health professionals are better able to inform patients about what to expect after encephalitis and provide appropriate referrals. The pathway could be developed as part of revisions of the National Guidelines for the management of suspected viral encephalitis, which are currently being planned [3, 14]. To ensure the effectiveness of this pathway, there should be further consultations between patient groups like *The Encephalitis Society* and *Headway*, health professionals, and researchers. This would begin the process of generating practical ways for tackling the current inadequacies around provision of care, and ensure that pathways of support are informed by the everyday reality facing people after encephalitis. In so doing, we will ensure a more holistic approach to improving the outcomes of encephalitis, one in which the entire trajectory (acute and chronic) of this devastating condition is fully addressed.

There are a number of limitations to this study and routes for further research. First, the majority of people who participated in this study had more moderate problems following encephalitis, in that most were able to discuss their experiences. Only a handful of cases had been severely affected to the extent that they could not understand enough about the research to take part. As such, the results of this study may not reflect the experiences of people who are left more severely disabled after encephalitis, such as people who are unable to talk. However for those people who are severely affected, recognition of the problems is typically easier, and there may be better provisions of care.

Second, as we noted in a previous publication, the small number of paediatric cases in this study means it is difficult to generalise our conclusions to the experiences of children and their parents [24]. Differences in paediatric care, and the fact that children are in education, may mean that the problems experienced are quite different from those of adult patients. It is important that there is further research to examine the challenges for children following encephalitis. Third, the study was conducted within the context of a single healthcare system: the NHS in the UK. This means that the findings may not be generalisable to other national settings, in which care pathways, and therefore peoples’ experiences of healthcare, will likely differ. Finally, since only a small number of cases in our study received rehabilitation after hospital we were unable to examine the processes involved in this care. Since our results highlight the importance of rehabilitation for recovery, future ethnographic research is needed to examine rehabilitation from the perspective of both patients and healthcare staff.

## Conclusion

This paper has documented the challenges which face people once they return home after HSV encephalitis. In particular, we have shown how problems like memory and language loss lead to the fragmentation of peoples’ every day, social worlds. What is important to understand, however, is that these problems do not play out in isolation of wider social contexts, like access to healthcare. As we have shown, peoples’ suffering in the wake of encephalitis is made worse through lack of access to appropriate information about, and care for, the chronic consequences of encephalitis. Improving outcomes in HSV encephalitis is therefore not only about creating the conditions for more timely diagnoses and better acute management. If we are to fully intervene in this devastating disease, we need to improve the systems of communication and care across the entire trajectory of encephalitis, which means extending support outwards, from the ward into the home.
